# Policy Interventions to Prevent Periodontal Disease in Rural India: A Scoping Review

**DOI:** 10.1155/ijod/6692835

**Published:** 2026-07-15

**Authors:** Nandini Nagpal, Neetha J. Shetty, Shruti Singh, Gitanjali P.

**Affiliations:** ^1^ Department of Periodontology, Manipal College of Dental Sciences Mangalore, Manipal Academy of Higher Education, Manipal, Karnataka, India, manipal.edu

**Keywords:** health policy, oral health promotion, periodontal disease, prevention, primary healthcare, public health dentistry, rural health centers

## Abstract

**Background:**

Periodontal diseases remain a major public health concern, particularly in rural populations where access to preventive oral healthcare is limited. Various periodontal disease prevention policies have been implemented through rural health centers, yet their effectiveness has not been comprehensively mapped.

**Objective:**

The objective of this study is to systematically map and evaluate existing evidence on the effectiveness of periodontal disease prevention policies implemented in rural health centers.

**Methods:**

This scoping review followed the Joanna Briggs Institute (JBI) methodology and PRISMA Extension for Scoping Reviews (PRISMA‐ScR) guidelines. Studies evaluating periodontal disease prevention policies in rural health settings were included. Databases including PubMed, Scopus, and Embase were searched. Data were charted and synthesized narratively.

**Results:**

Nineteen studies met the inclusion criteria. Most studies evaluated community‐based oral health education programs, training of frontline health workers, screening initiatives, and outreach activities in rural India. Short‐term improvements were observed in oral health knowledge, oral hygiene practices, plaque levels, and community engagement. However, evidence regarding long‐term periodontal outcomes, policy implementation, sustainability, and cost‐effectiveness was limited.

**Conclusion:**

Existing evidence suggests that community‐based oral health education programs, outreach activities, and training of frontline health workers can improve oral health knowledge, hygiene practices, and short‐term periodontal outcomes in rural India. However, evidence evaluating formal periodontal disease prevention policies, their long‐term effectiveness, sustainability, and cost‐effectiveness remains limited. These findings highlight the need for stronger integration of periodontal disease prevention into primary healthcare services, continued capacity building of community health workers, and robust policy evaluation frameworks. Future research should focus on long‐term outcomes and scalable implementation models to support evidence‐based oral health policy development in rural India.

## 1. Introduction

Periodontal diseases, including gingivitis and periodontitis, are among the most common chronic inflammatory diseases affecting oral health worldwide. Gingivitis is characterized by reversible inflammation of the gingival tissues, whereas periodontitis results in the progressive destruction of the supporting structures of the teeth and may ultimately lead to tooth loss [1, 2]. In addition to their oral consequences, periodontal diseases have been associated with systemic conditions such as diabetes mellitus [3], cardiovascular disease [4], and adverse pregnancy outcomes [5]. Severe periodontitis is recognized as a major global public health problem and remains one of the most prevalent chronic diseases worldwide.

In India, periodontal disease remains highly prevalent across all age groups. National surveys and regional studies have consistently demonstrated a substantial burden of gingivitis and periodontitis, particularly among socioeconomically disadvantaged populations [6, 7]. Rural communities often experience poorer periodontal health outcomes than metropolitan populations because of reduced access to dental services, lower oral health literacy, financial constraints, and shortages of trained oral health professionals [6, 7].

Prevention of periodontal disease primarily relies on effective plaque control, oral health education, tobacco cessation, regular dental visits, and early identification of the disease [8]. Treatment approaches include professional plaque and calculus removal, scaling and root planing, supportive periodontal therapy, and long‐term maintenance care. Because periodontal diseases are largely preventable, public health strategies and preventive policies can play an important role in reducing the disease burden at the population level [9–12].

Internationally, oral health promotion has increasingly been incorporated into public health frameworks through school‐based programs, community prevention initiatives, workforce development strategies, and integration of oral health into primary healthcare systems [9, 12]. The WHO Global Oral Health Action Plan 2023–2030 emphasizes the integration of oral health within universal health coverage and primary healthcare services [9]. In India, oral health promotion activities have been supported through the National Oral Health Program (NOHP), National Health Mission initiatives, school oral health programs, and community‐based services delivered through primary health centers (PHCs), community health centers (CHCs), accredited social health activist (ASHA) workers, and Anganwadi workers [9–11].

Preventive oral health policies play a vital role in reducing the burden of periodontal disease by shifting the focus from treatment to prevention and health promotion [9–12]. These policies are developed within broader public health frameworks, informed by epidemiological evidence, population health needs, and available resources [9, 12]. Within these frameworks, preventive interventions—such as community‐based oral health programs, school oral health initiatives, workforce training, and preventive care guidelines—serve as practical strategies to achieve policy objectives [9–12]. Understanding the relationship between policies and the interventions they support is essential for evaluating how preventive strategies are designed, implemented, and sustained. However, the successful implementation of these policies depends on several contextual factors, including health system capacity, governance, resource availability, and community engagement [9–12]. These factors can either facilitate or hinder the translation of policy into effective practice.

The success of prevention policies and interventions depends on a number of factors that can either support or hinder their implementation. Facilitators include government commitment, adequate funding, trained health workers, community engagement, and integration of oral health services into existing healthcare systems [9–12]. Common barriers include workforce shortages, insufficient infrastructure, competing health priorities, limited awareness, and restricted access to care in geographically remote regions. Understanding these factors is important when evaluating the effectiveness of periodontal disease prevention policies and interventions [9–12].

Therefore, this scoping review aimed to map and synthesize the available evidence on periodontal disease prevention policies and policy‐related preventive interventions implemented in rural India. The review sought to identify the prevention approaches that have been used, explore implementation barriers and facilitators, examine reported outcomes, and identify gaps that may inform future research, practice, and policy development.

## 2. Review Questions

1. What periodontal disease prevention policies and policy‐related preventive interventions have been implemented in rural India?
2. What implementation approaches have been used to deliver these policies and interventions in rural healthcare settings?
3. What barriers and facilitators influence the implementation and effectiveness of periodontal disease prevention policies and interventions in rural India?
4. What outcomes have been reported to evaluate the effectiveness of these policies and interventions?
5. What evidence gaps exist in the current literature on periodontal disease prevention policies and interventions in rural India?

## 3. Methods

This scoping review followed the Joanna Briggs Institute (JBI) methodology for scoping reviews [13] and was reported according to PRISMA Extension for Scoping Reviews (PRISMA‐ScR) guidelines [14]. This review protocol was prospectively registered on the Open Science Framework (OSF) platform (Registration DOI: 10.17605/OSF.IO/M63WP). The registration included details of the study design, eligibility criteria, and planned methods for data extraction and analysis.

### 3.1. Eligibility Criteria (PCC Framework)

#### 3.1.1. Population (P)

Rural populations in India, including children, adolescents, adults, and older adults, were targeted by periodontal disease prevention programs, oral health promotion initiatives, or policy‐related preventive interventions. All age groups were included because periodontal disease prevention begins early in life through the establishment of healthy oral hygiene behaviors and continues throughout adulthood to prevent disease progression and tooth loss [8].

#### 3.1.2. Concept (C)

Periodontal disease prevention policies and policy‐related preventive interventions are implemented through public health, primary healthcare, educational, or community‐based settings. This included oral health education programs, community outreach initiatives, workforce training programs, school‐based oral health activities, and preventive strategies delivered within broader public health frameworks [9–11].

#### 3.1.3. Context (C)

Rural healthcare and community settings in India include PHCs, CHCs, schools, tribal communities, outreach programs, and community‐based health services.

### 3.2. Inclusion and Exclusion Criteria

**Table:** 

Domain	Inclusion criteria	Exclusion criteria
Population	Children, adolescents, adults, and older adults residing in rural India who were targeted by periodontal disease prevention programs, oral health promotion activities, or policy‐related preventive interventions	Urban‐only populations, animal studies, laboratory studies
Concept	Periodontal disease prevention policies and policy‐related preventive interventions, including oral health education programs, community outreach initiatives, school‐based oral health activities, workforce training programs, and preventive strategies delivered through public health systems	Studies focused solely on diagnosis, treatment, surgical periodontal procedures, implant therapy, or other interventions unrelated to periodontal disease prevention
Context	Rural healthcare and community settings in India, including primary health centers (PHCs), community health centers (CHCs), schools, tribal communities, outreach programs, and community‐based services	Studies conducted exclusively in urban settings, tertiary care hospitals, or specialist clinics without a rural/community prevention component
Study types	Original research studies, program evaluations, implementation studies, observational studies, quasiexperimental studies, and relevant policy or government reports	Editorials, commentaries, letters to the editor, conference abstracts, opinion pieces, narrative reviews, systematic reviews, and protocols
Language	English‐language publications	Non‐English publications
Availability	Full‐text articles available for review	Articles with unavailable full text

### 3.3. Information Sources

Databases including PubMed, Scopus, and Embase were searched, and WHO reports were screened. Government reports were sourced from publicly accessible websites, including the Ministry of Health and Family Welfare (Government of India), National Health Mission resources, and oral health program documents.

### 3.4. Search Strategy

To ensure comprehensive coverage of the broad literature, no data restrictions were applied. MeSH terms and keywords including periodontal disease, periodontitis, periodontal health, gingivitis, rural health centers, and prevention policies were used with appropriate Boolean operators (Table 1).

**Table 1:** Search string generated using various databases.

**PubMed** search strategy	(“Periodontal Diseases” [MeSH] OR periodontitis OR gingivitis) AND (“Primary Prevention” [MeSH] OR prevention OR “health policy” OR “public health program”) AND (“Rural Health Services” [MeSH] OR “Rural Population” [MeSH] OR rural health centre OR primary health centre
**Scopus** uses TITLE‐ABS‐KEY instead of MeSH terms	(TITLE‐ABS‐KEY (“periodontal disease” OR periodontitis OR gingivitis)) AND (TITLE‐ABS‐KEY (“primary prevention” OR prevention OR “health policy” OR “public health policy OR “oral health program ^∗^ ” OR “community oral health” OR “health promotion”)) AND (TITLE‐ABS‐KEY (“rural health service ^∗^” OR “rural health centre ^∗^ ” OR “primary health centre ^∗^” OR PHC OR “community health centre ^∗^ ” OR “rural population”)) AND (PUBYEAR > 1999) AND (LIMIT‐TO (LANGUAGE, “English”))
**Embase** uses Emtree terms with /exp for exploded terms	(‘periodontal disease’/exp OR periodontitis:ti,ab OR gingivitis:ti,ab) AND (‘disease prevention’/exp OR ‘health policy’/exp OR prevention:ti,ab OR ‘public health program’:ti,ab OR ‘oral health promotion’:ti,ab) AND (‘rural health service’/exp OR ‘rural population’/exp OR ‘primary health care’/exp’primary health centre’:ti,ab OR ‘community health centre’:ti,ab) AND [2000–2026]/pyAND[english]

*Note:* Bold indicates the database headings.

### 3.5. Study Selection

All identified records were exported to Rayyan AI for screening, and duplicate records were removed prior to title and abstract screening [15]. Rayyan is a web‐based platform designed to facilitate screening and study selection during evidence synthesis projects by supporting independent reviewer screening and conflict resolution [15]. Two independent reviewers (Nandini Nagpal and Neetha J. Shetty) screened titles and abstracts, and full‐text screening was conducted for eligible articles. Disagreements were resolved through discussion. A PRISMA flow diagram illustrated the selection process [14].

### 3.6. Data Charting

A standardized data extraction form was developed, including author and year, study design, type of policy/intervention, target population, implementation strategy, duration of intervention, outcome measures, key findings, and reported effectiveness (Table 2).

**Table 2:** Data interpretation of the various studies included.

Author and year	Study design	Type of policy/intervention	Target population	Implementation strategy	Duration of intervention	Outcome measures	Key findings	Reported effectiveness
Shah et al., 2017 [6]	Cross‐sectional survey	Prevalence assessment (no intervention)	5–7, 12–15, 35–44, 65–74 years; rural Haryana	Multistage cluster sampling, trained examiners	N/A	Deft/DMFT, CPI, LOA, mucosal lesions	Caries 31%–65%; periodontal disease 65%–90% by age group	Feasible for national surveys
Mehta and Kaur, 2012 [7]	Cross‐sectional	Not specified (assessment for future promotion program)	12‐year‐old rural schoolchildren	Self‐administered questionnaire under supervision	N/A	Oral health knowledge, attitudes, and practices; dental history	Poor daily cleaning; low dentist visits; low fluoride/sugar awareness; gender differences	N/A
Mehta et al., 1987 [16]	Not specified	Dental prophylaxis (scaling + tooth brushing instructions)	Rural children and factory workers	Professional scaling + public health brushing instructions	Yearly/half‐yearly/quarterly (not fully specified)	Prevalence of gingivitis/periodontal disease; calculus; plaque	High baseline prevalence; significant reductions with treatment; more frequent better	Considerable reduction
Asawa et al., 2015 [17]	Retrospective	Community outreach programs	Individuals 4–80 years	Conducted by dental college/hospital	2 years	Patient numbers; disease patterns (caries, periodontal, fluorosis); services; referrals	High attendance; common diseases; improved referrals after guidelines	Improved after strategies
Kaur et al., 2014 [18]	Cross‐sectional	Not specified (observational + oral health education)	School children 5, 12, and 15 years	Clinical exams, questionnaire, education via presentations/pamphlets	Not specified	Gingival index; gingivitis prevalence; oral hygiene practices	Higher gingivitis in rural; linked to brushing frequency; lower rural awareness	N/A
Gupta and Kaur, 2012 [19]	Cross‐sectional	Not specified	15‐year‐old schoolchildren	Not specified	N/A	Oral health knowledge/attitude/practices; diet score	High knowledge in some areas, low fluoride; linked to residence or school type	N/A
Kumar et al., 2009 [20]	Cross‐sectional	Not specified	Bhil tribal adults 15–54 years	Not specified	Not specified	OHI‐S; DMFT/DMFS; treatment needs; CPI	Scores ↑ with age; high needs; pockets in older groups	N/A
Kumar et al., 2008 [21]	Not specified	Not specified	Male green marble mine laborers	Not specified	N/A	Periodontal disease prevalence; bleeding; calculus, healthy sextants; pockets	High prevalence (98.2%); widespread bleeding/calculus; age‐related destruction	N/A
Shahi et al., 2019 [22]	Not specified	Not specified (oral health behavior assessment)	Adolescent tobacco consumers	Self‐administered questionnaire by trained investigator	N/A	Hygiene aids; brushing frequency; dental visits; tobacco patterns	Toothbrush/toothpaste common; media influence; low interdental aids/visits	N/A
Salunke et al., 2019 [23]	Cross‐sectional	Not specified	Elderly 60+ years	Dental exam + verbal interview	N/A	Caries prevalence; DMFT; hygiene practices; pain; treatment‐seeking; denture use	High caries (76.4%); low brush/paste use linked to higher DMFT; high pain but low visits	Lower DMFT with brush/paste use
Sadana et al., 2017 [24]	Double‐blinded RCT (field trial)	Oral health education (lectures, pamphlets, AV aids)	School children 10–12 years, Amritsar, Punjab	Random assignment to four groups in classrooms	6 weeks	Plaque index (Silness and Löe); oral health knowledge questionnaire	Knowledge ↑ in all groups (highest pamphlets); plaque ↓	Highly effective; pamphlets + AV additive to verbal
Khanna et al., 2021 [25]	Quasiexperimental (pre–post)	Training of Anganwadi/ASHA workers on oral hygiene	Children 1–6 years (tribal Rajasthan) + 87 workers	Lectures, videos, posters, demos, local language	6 months	DMFT/deft, OHI‐S/OHI‐S‐M, plaque index, Oratest, brushing/rinsing	↑ Brushing/rinsing; ↓ debris/calculus/plaque/OHI/caries activity	Effective; ripple effect via workers
Potlia et al., 2021 [26]	Two‐stage intervention trial	Animated‐movie infotainment ± parental orientation/SMS	6‐year‐old children + mothers, Davangere	Classroom sessions (twice monthly) + parental/SMS	2 months	Modified plaque index; knowledge/attitude questionnaires	Plaque ↓ (max with parental); knowledge/attitude ↑ in children and mothers	Appreciable with parental involvement
Singh et al., 2020 [27]	Cross‐sectional	None (association of oral health literacy with periodontal status)	Low‐income dental institute workers (class III/IV), Patna	REALD‐30 literacy test + periodontal exam	N/A	Periodontal status; REALD‐30 score; hygiene habits	Low literacy → severe periodontitis	Improving literacy reduces burden
Balasupp ramaniem et al., 2017 [28]	Prospective cohort	Initial periodontal therapy + hygiene motivation	Young adults 18–26 years, rural Tamil Nadu	Tell–show–do + single phone reinforcement	6 months	Modified Quigley–Hein plaque, gingival index, MPBI, self‐efficacy	Significant ↓ plaque/GI/MPBI motivation correlated with bleeding reduction	Effective in rural young adults
Cyriac et al., 2023 [29]	Cross‐sectional descriptive	None (KAP assessment + one‐time education)	Government primary‐school teachers, Faridabad	Supervised questionnaire + brief education	N/A	Knowledge, attitude, and practice scores	Good knowledge and moderate attitude/practice; females higher	Teachers need better training
Kumar et al., 2023 [30]	Expert consensus guidelines	SP GCP Recommendations for gum care (education, PMPR, SPT)	General Indian population (all ages with teeth)	Patient education, mechanical plaque removal, risk control, lifelong recall	Lifelong (recall 3–12 months)	BOP less than 10%, plaque and gingival indices, tooth loss	High prevalence (15%–96%); gingivitis reversible; periodontitis needs maintenance	Effective with daily hygiene + SPT
Shingare et al., 2025 [31]	Pre–post intervention	Structured awareness + supervised brushing Anganwadi workers	Anganwadi workers + children 3–5 years, rural Maharashtra	Dentist training + daily supervised brushing	3 months	OHI‐DI/debris index simplified	OHI‐DI ↓ from 0.94 to 0.54 (intervention) vs. stable control	Highly effective and cost‐effective via workers
Rahmath Meeral et al., 2024 [32]	Cross‐sectional epidemiological	None (descriptive assessment)	Kanikkaran seminomadic tribes, Mundandhurai Hills, TN	WHO form + convenience sampling	N/A	DMFT, periodontal status, premalignant/noncarious lesions, sugar/tobacco use	High caries/periodontal/premalignant risk; education + occupation	Urgent need for awareness/services

Abbreviations: ASHA, accredited social health activist; BOP, bleeding on probing; CHC, community health center; CPI/CPITN, Community Periodontal Index/Community Periodontal Index of Treatment Needs (if used); GI, gingival index; OHI, Oral Hygiene Index; PHC, primary health center; PI, plaque index.

## 4. Results

### 4.1. Study Selection

A total of 57 records were identified through database searching. No additional records were identified from other sources. Following the removal of duplicates, 57 records remained for title and abstract screening. During screening, 29 records were excluded because they did not meet the inclusion criteria, including studies conducted exclusively in urban settings, hospital‐based studies, and articles unrelated to periodontal disease prevention policies or preventive interventions. Consequently, 28 reports were sought for retrieval, and all were successfully obtained and assessed for eligibility. Following the full‐text review, 19 studies met the eligibility criteria and were included in the final scoping review.

Of the 19 studies included in this review, 18 were primary research studies evaluating the implementation and effectiveness of periodontal disease prevention interventions, and one was an expert consensus guideline. No formal government policy documents or policy evaluation studies met the inclusion criteria. Therefore, although this review sought evidence on periodontal disease prevention policies and interventions, the available literature consisted almost entirely of intervention studies rather than formal policy documents. Consequently, the findings primarily reflect evidence on policy‐related preventive interventions rather than evaluations of formal periodontal disease prevention policies.

**Figure 1:**
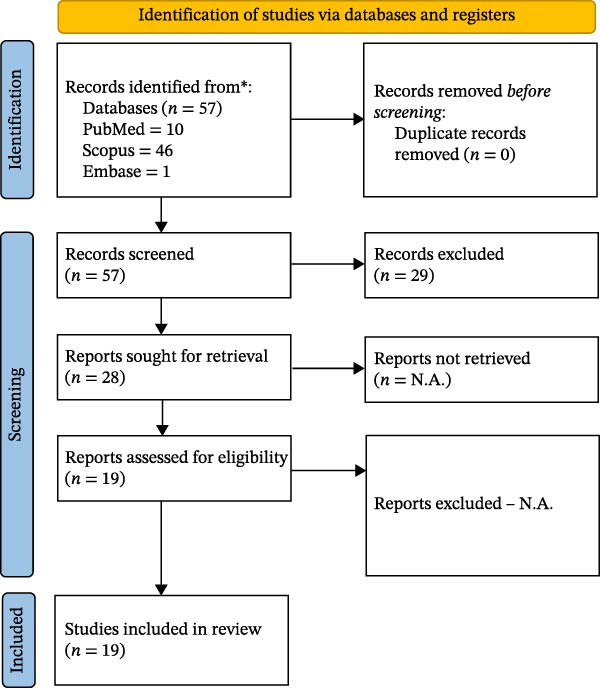
PRISMA flow diagram.

The study selection process is illustrated in the PRISMA 2020 flow diagram (Figure 1).

### 4.2. Characteristics of Included Studies

Table 2 summarizes the characteristics of the included studies, including the study design, population, intervention, setting, and key findings.

#### 4.2.1. Where the Studies Came From

All 19 studies were conducted in India —mostly in rural or tribal regions across different states [7, 16–32].

#### 4.2.2. Designs Used

The included evidence consisted predominantly of cross‐sectional surveys [7, 16–23, 32], program evaluations, intervention studies, and descriptive reports conducted in rural or tribal populations [17, 24–29, 31]. Several studies evaluated oral health education programs, community outreach activities, training of frontline health workers, and behavioral interventions, while others assessed periodontal disease burden, oral health knowledge, attitudes, and practices (KAP) [17, 19, 24–26, 29, 31]. One included guideline publication provided expert consensus recommendations for periodontal care but did not evaluate policy implementation [30]. Notably, very few studies evaluated formal government policies, and no large‐scale policy implementation studies conducted within routine rural primary healthcare systems were identified [7, 16–32].

#### 4.2.3. Main Types of Prevention Approaches

When grouped by actual implementation, six broad categories emerged: education programs through community meetings, school sessions, and demonstrations by health workers or dentists [19, 22, 23, 28, 30, 32]; integration of basic gum screening and advice into routine primary health care services [18, 26, 32]; periodic screening camps using the Community Periodontal Index (CPI) or similar indices [7, 20, 25, 31, 32]; training frontline workers (such as ASHAs and Anganwadi staff) to recognize early signs and refer cases [26, 31]; inclusion of tobacco cessation messages or counseling in oral health promotion [23]; and occasional mobile dental outreach to remote areas [7].

### 4.3. What Outcomes Were Measured

#### 4.3.1. Clinical Markers

The CPI was the most frequently used clinical measure [18, 20, 25, 28, 32]. Other common indices included the gingival index (GI), plaque index (PI), and bleeding on probing (BOP) [28, 30–32].

#### 4.3.2. Behavioral and Knowledge Markers

Studies commonly assessed self‐reported brushing frequency and other oral hygiene habits [19, 22, 23, 27, 29, 30]. KAP questionnaires were used to evaluate educational interventions [19, 23, 28, 30]. Changes in tobacco use were evaluated as part of oral health promotion efforts [26]. Some studies also measured whether participants utilized dental services or followed through on referrals [21, 29, 32].

### 4.4. Effectiveness of Periodontal Disease Prevention Interventions

#### 4.4.1. Awareness, Behavior Change, and Short‐Term Clinical Effects

Education‐oriented projects consistently reported short‐term gains in knowledge, self‐reported brushing habits, and —when parents/caregivers were involved —noticeable reductions in plaque scores [28, 30, 32]. Several studies also showed reductions in inflammation and plaque, though changes in the CPI were generally modest [28, 30, 32].

#### 4.4.2. Long‐Term Sustainability and Limitations

Follow‐up rarely extended beyond 6–12 months, which is concerning given the slow progression of periodontal disease and the high risk of relapse once support ends [28, 30, 32]. Virtually, no studies provided long‐term data on attachment levels, tooth survival, or sustained clinical improvements.

#### 4.4.3. Integration Into Routine Primary Care and Community‐Based Approaches

Efforts to embed basic oral health activities (screening, advice, and referrals) into routine PHC services achieved higher screening coverage, earlier gingivitis detection, and improved referral flow [21, 29, 32]. Training ASHAs and Anganwadi workers enhanced early recognition, community trust, and engagement [26, 32]. Mobile dental outreach successfully reached remote villages [21]. However, persistent barriers included insufficient trained staff, unreliable equipment, delayed budgets, infrequent visits, and a frequent shift in focus from prevention to curative treatment of pain or obvious decay rather than building lasting preventive habits [21, 29, 32].

## 5. Discussion

This scoping review mapped the available evidence on periodontal disease prevention policies and policy‐related preventive interventions in rural India. Of the 19 included studies, 18 were primary research studies evaluating preventive interventions, and one was an expert consensus guideline. No formal government policy documents or policy evaluation studies met the inclusion criteria. Consequently, the available evidence primarily reflects the implementation and effectiveness of policy‐related preventive interventions rather than formal evaluations of periodontal disease prevention policies. Overall, the included studies demonstrated that rural periodontal disease prevention efforts predominantly focused on oral health education programs [19, 24, 26, 29], community outreach programs [17], screening activities [16, 21], and training of frontline health workers [25, 31] rather than formal policy implementation. The review also identified considerable variation in implementation strategies and outcome measures, limiting direct comparison across studies [7, 16–32].

Regarding the first research question, no formal government policy documents or policy evaluation studies met the inclusion criteria. Instead, the available literature consisted almost entirely of primary research studies evaluating policy‐related preventive interventions, including oral health education programs [19, 24, 26, 29], supervised toothbrushing initiatives [24, 26], outreach camps [17], integration of oral health into primary healthcare [25, 31], and capacity‐building activities involving ASHA and Anganwadi workers [25, 31]. This finding highlights the limited evidence on the development, implementation, and evaluation of formal periodontal disease prevention policies in rural India.

With respect to implementation strategies, the most reported approaches included school‐based oral health education [19, 24, 26], community awareness programs [19, 23, 29], integration of oral health promotion into existing healthcare activities [25, 31], and training of frontline healthcare personnel [25, 31]. These strategies aimed to improve oral health knowledge, encourage positive oral hygiene behaviors, and facilitate the early identification and referral of periodontal conditions [19, 28–30, 32].

The review further demonstrated that effectiveness was primarily assessed using clinical indicators such as plaque scores, gingival indices, and CPI scores [16, 21, 28, 32], together with behavioral outcomes including oral hygiene practices, oral health knowledge, and healthcare‐seeking behavior [18, 19, 22–24, 26, 29]. Although many interventions reported short‐term improvements in knowledge and oral hygiene practices [19, 24, 26, 29] and plaque control [24, 26, 28, 32], evidence supporting sustained long‐term periodontal health benefits was limited. Finally, several important gaps were identified, including a lack of policy‐level evaluations, limited long‐term follow‐up, absence of economic analyses, and insufficient evidence regarding large‐scale integration of periodontal prevention within routine primary healthcare systems [7, 16–32].

These findings are consistent with broader oral health promotion literature, which has demonstrated that educational and behavioral interventions can improve oral health knowledge and short‐term oral hygiene practices [19, 23, 28, 30, 32]. Similar challenges relating to workforce limitations, sustainability, and integration into primary healthcare systems have been reported globally [9–12]. The WHO Global Oral Health Action Plan 2023–2030 likewise emphasizes the integration of oral health within primary healthcare systems as a key strategy for reducing oral health inequalities and improving population‐level outcomes [9].

Weaving basic oral exams and guidance into everyday PHC services seemed practical and hopeful in theory, as it could capitalize on established systems for regular screening, timely referrals, and ongoing reinforcement of prevention reminders [9–11]. In reality, however, familiar roadblocks persisted across the studies: ongoing staff shortages (with scarce dental experts or even general health workers trained for oral care tasks), persistent shortages of essential tools and materials at PHC sites, and funding that invariably dried up after the pilot phase ended [17, 21, 25, 31, 32]. This led to patchy rollout, poor long‐term sustainability, and recurring documentation of these same hurdles without proven, scalable solutions for building enduring programs [21, 29, 32].

In summary, the studies painted a clear picture of encouraging surface‐level tactics that excelled at boosting awareness but struggled to demonstrate lasting clinical effects or surmount core systemic issues [19, 24, 26, 28, 29, 32]. This pattern underscored the pressing need for future rural strategies to emphasize extended evaluations, deeper integration with PHC (backed by dedicated funding and training), and innovative approaches that directly tackled behavior retention and structural barriers [9–11].

### 5.1. Strengths and Limitations

This review has several strengths. It followed the JBI methodology for scoping reviews and was reported according to the PRISMA‐ScR guidelines. Multiple electronic databases were searched using a predefined search strategy, and study selection was conducted independently by two reviewers. The review provides a comprehensive overview of the available evidence relating to periodontal disease prevention interventions and policy‐related activities in rural healthcare settings.

However, several limitations should be acknowledged. Only English‐language publications were included, which may have resulted in the omission of relevant studies published in other languages. In addition, the available evidence was derived almost exclusively from India, limiting the generalizability of the findings to other low‐resource settings. Most included studies evaluated preventive interventions rather than formal policy implementation, making it difficult to draw conclusions regarding the policy effectiveness. Furthermore, substantial heterogeneity in study designs, interventions, and outcome measures limited direct comparison across studies, and the methodological quality of included studies was not formally assessed.

## 6. Evidence Gaps

Follow‐up periods in the included studies were rarely extended beyond 6–12 months [24, 26, 28, 31, 32], despite the chronic, lifelong nature of periodontal disease and the well‐recognized risk of relapse once interventions cease [8]. This short‐term focus severely limits the understanding of sustained clinical benefits, such as long‐term reductions in attachment loss, tooth retention, or progression to advanced periodontitis.

Most of the available evidence was derived from small‐scale, local pilot projects or NGO‐led initiatives rather than large‐scale, formally implemented, and sustained state‐ or national‐level policies [17–32]. There was a notable absence of evaluations of scaled‐up integration within India’s primary healthcare system (e. g., under the NOHP or Ayushman Bharat), including challenges in sustainability, replication, and system‐wide impact [9–11].

No included study conducted a formal cost‐effectiveness analysis, despite the resource constraints of rural primary care systems. Given the economic implications (e. g., productivity losses and treatment costs far exceeding preventive investments), this represented a major gap for informing policy prioritization and resource allocation.

The evidence base was exclusively Indian, with studies concentrated in certain rural or tribal regions [7, 16–32]. This left certain critical questions unanswered about the transferability and adaptation of these approaches to other low‐resource rural settings—such as tribal areas in sub‐Saharan Africa or other geographically and culturally diverse low‐ and middle‐income contexts —where infrastructure, workforce availability, cultural factors, and systemic barriers may differ substantially.

Addressing these gaps through longer‐term studies, rigorous economic evaluations, scaled policy implementations, and comparative research across diverse settings would substantially strengthen the evidence for effective, sustainable periodontal disease prevention in rural primary healthcare [9–11].

## 7. Conclusion

This scoping review identified 19 studies examining periodontal disease prevention interventions and policy‐related activities in rural India.

The available evidence suggests that oral health education programs improved oral health knowledge and oral hygiene behaviors [19, 24, 26, 29], community outreach initiatives enhanced access to preventive oral health services [17], and training of frontline health workers supported community‐based oral health promotion [25, 31]. Several interventions also reported improvements in selected short‐term clinical outcomes, including plaque control and oral hygiene status [24, 26, 28, 32]. However, the evidence regarding the effectiveness of formal periodontal disease prevention policies remains limited.

Important gaps remain in relation to long‐term effectiveness, cost‐effectiveness, scalability, and integration within routine primary healthcare systems. Future research should prioritize rigorous evaluations of the implementation of the WHO Global Oral Health Action Plan, the NOHP, and the National Health Mission, together with longer follow‐up periods, economic analyses, and sustainable models of integration within rural healthcare services [9–11].

Strengthening the evidence base in these areas will support the development of effective and sustainable approaches to reducing the burden of periodontal disease in rural populations.

## Author Contributions

**Nandini Nagpal:** conceptualization, methodology, investigation, software, resources, data curation, writing – original draft preparation, writing – review and editing, visualization, project administration. **Neetha J. Shetty**: methodology, investigation, validation, formal analysis, supervision, writing – original draft preparation, writing – review and editing, visualization. **Shruti Singh**: investigation, data curation, validation, writing – review and editing, assisted in literature screening, study selection, data extraction, organization of study characteristics. **Gitanjali P.**: data curation, validation, writing – review and editing, assisted in data verification, synthesis of extracted information, formatting of tables and references, final manuscript editing.

## Funding

This study did not receive any specific funding from public, commercial, or not‐for‐profit funding agencies.

## Disclosure

All the authors have read and agreed to the published version of the manuscript. All machine‐assisted content was reviewed and verified by the authors. All authors have read and approved the final version of the manuscript. Neetha J. Shetty as the corresponding author and manuscript guarantor had full access to all of the data in this study and takes complete responsibility for the integrity of the data and the accuracy of the data analysis.

## Ethics Statement

Ethical approval was not required for this study because it was a scoping review based exclusively on the published literature and publicly available documents. No human participants were recruited, no primary data were collected, and no identifiable personal information was accessed.

## Conflicts of Interest

The authors declare no conflicts of interest.

## Supporting Information

Additional supporting information can be found online in the Supporting Information section.

## Supporting information


**Supporting Information** Supporting File S1: PRISMA Extension for Scoping Reviews (PRISMA‐ScR) Checklist indicating where each reporting item is addressed in the manuscript.

## Data Availability

No new data were generated. All data were obtained from published sources available in public databases (PubMed/MEDLINE, Web of Science, Scopus, and Embase) and via the articles cited. The authors confirm that the data supporting the findings of this study are available within the article.
